# The Effect of Eco-Friendly Inhibitors on the Corrosion Properties of Concrete Reinforcement in Harsh Environments

**DOI:** 10.3390/ma15144746

**Published:** 2022-07-06

**Authors:** Rui’E Guo, Qian Zhang, ZaiXing Wang, Morteza Tayebi, Bejan Hamawandi

**Affiliations:** 1 College of Science and Engineering, Xi’an Siyuan University, Xi’an 710038, China; zaixingwang2@gmail.com; 2 School of Architecture and Rail Transit, Xi’an Vocational and Technical College, Xi’an 710077, China; qian.zhang10@gmail.com; 3 Young Researchers and Elites Club, Science and Research Branch, Islamic Azad University, Tehran 14778-93855, Iran; morteza.tayebi@srbiau.ac.ir; 4 Department of Applied Physics, KTH Royal Institute of Technology, SE-106 91 Stockholm, Sweden

**Keywords:** green inhibitor, reinforced concrete, guar gum, Arabic gum, corrosion

## Abstract

In the present research, the synergistic effect of Arabic and guar gum inhibitors on the corrosion efficiency of concrete reinforcement was investigated. Thus, eight types of Arabic and guar gum combinations with 100, 250, 500, 750, and 1000 ppm were added to the steel reinforcement for 1, 7, 28, 48, and 72 days. The corrosion behavior of the samples was investigated by the electrochemical impedance (EIS) test. Water transmissibility, electrical resistivity, and compressive strength of concrete were also studied. The results showed that adding inhibitors generally increased the compressive strength of concrete. It was also found that water transmissibility was reduced by the addition of inhibitors. The electrical resistivity of the samples increased slightly with increasing time up to 72 days. EIS and Tafel results have demonstrated that Arabic and guar gums are effective inhibitors for reinforced concrete structures. Furthermore, scanning electron microscopy (SEM) and Fourier transform infrared spectroscopy (FTIR) utilized to analyze the samples indicated that inhibitor grain size was enhanced by enhancing the concentration of the inhibitor combination, showing that the guar and Arabic inhibitor combinations were properly absorbed on the reinforcement surface. Results showed that a sample with 250 ppm Arabic gum and 250 ppm guar gum having a properly distributed inhibitor combination on the reinforcement surface creates a desirable cathode current.

## 1. Introduction

In recent years, increasing the life of reinforced concrete structures, particularly the marine structures, has been one of the main concerns of researchers and engineers in the construction industry [1,2,3,4]. To avoid the destruction of concrete structures the use of corrosion inhibitors has been widely demonstrated as an efficient technique [5,6]. Generally, the inhibitors are chemical materials which decrease the corrosion rate of concrete reinforcements without dramatically changing the concentration of corrosion agents [7,8,9]. These substances are mostly cost effective (given their low required concentrations) and easy to use (in comparison with other corrosion protection methods). Among them, migrating corrosion inhibitors are typically either dissolved or applied in a liquid state on the surface of the concrete [10].

Corrosion of reinforcing steel rebars in concrete is an electrochemical process which occurs prematurely through the Cl^−^ ion penetration in carbonate environments when there are different concentrations of dissolved ions in the concrete. Corrosion cells are created by the flow of ions and electrons between the anodic and cathodic regions. In the cells, steel rebars act as electrical conductors, while the concrete fluid matrix acts as the electrolytic environment into which ions move [11]. The presence of different electrochemical potentials which form corrosion cells is the result of chemical or physical heterogeneity at the metal surface. The reason for potential differences between different spots of rebar is the variation in moisture, aeration, and stress concentration in the concrete structure. Consequently, the formation and accumulation of the corrosion products degrade the concrete structure and reduce its service life [12,13].

Therefore, this concept eliminates other methods for corrosion protection and surface protective mechanisms, e.g., using mineral additives and pore blockers that change the concentration of invasive agents. Corrosion inhibitors can behave in two ways during the corrosion process, delaying the loss of the reinforcement, strengthening the compact passive film, or reducing the corrosion rate after depassivation [14]. Hence, the use of corrosion inhibitors with sufficient concentrations hinders the corrosion process. Recently, due to the increasing concern regarding the permanence of reinforced concrete structures, the use of inhibitors has gradually enhanced. To protect reinforced concrete structures, various methods have been developed. For newly constructed structures, inhibitors may be added to the concrete mixture to protect the structure against corrosion or postpone it. Inhibitors, especially those which are admixed with the concrete, can modify the fresh and hardened concrete properties and may affect other properties such as the strength and setting time [15].

MCIs are also used as surficial inhibitors. Both fixed and MCI can reduce the corrosion of steel rebars by forming a protective film on the rebar surface via the adsorption mechanism. For instance, alkanolamines, amines, and their salts are considered organic inhibitors. It has been reported that alkanolamine-based inhibitors increase corrosion resistance due to the carbonation effect, only for environments containing chloride ions [16,17,18]. Furthermore, natural organic compounds derived from different types of plants are also introduced as green inhibitors. These plants are enriched sources of naturally occurring corrosion inhibitor compounds, which are biodegradable and can be extracted through simple and inexpensive methods. Many investigations have been carried out on extracting organic inhibitors from stems, leaves, or seeds of plants such as Arabic gum and guar gum. Arabic gum is a multifunctional hydrocolloid with a natural branched chain structure that contains neutral or slightly acidic arabinogalactan proteins and is composed of arabinose and galactose monosaccharides containing calcium, magnesium, and potassium. Arabic gum is mainly composed of high molecular weight polysaccharides and Ca, Mg, and K salts, which during hydrolysis are decomposed into two main parts: polysaccharides and proteins. Chemically, Arabic gum is a complex combination of macromolecules with various sizes and compositions, a high carbohydrate content, and a low content of proteins. Arabic gum also contains amino acids. Omorn, et al. [19] investigated the inhibitory efficiency of Arabic gum in H_2_SO_4_ solution for carbon steel at different temperatures. The results of the weight loss test showed that the addition of Arabic gum increased the yield at a constant temperature. Additionally, the inhibitory effect of Arabic gum for AA1060 aluminum sheets was increased at 40 °C.

Chemically, guar gum is mainly composed of a high molecular weight polysaccharide namely galactomannan, in which galactose side chains are located on the main mannan chain. Typically, in the guar gum, approximately one galactose unit is considered for each of the mannose units [19]. This gum has a high viscosity, it is soluble at about 95 °C, and it is compatible with other hydrocolloids, carbohydrates, chemically modified starches, cellulose, and water-soluble proteins and/or proteins [20]. The inhibitory effect of guar gum as an environmentally friendly corrosion inhibitor for commercially pure aluminum HCl solution was evaluated. The results of weight loss, electrochemical polarization, FTIR, and SEM analyses confirmed the significant corrosion inhibition of guar gum for commercially pure aluminum [20]. In another study [21], guar gum was used as a corrosion inhibitor for steel in the H_2_SO_4_ solution. The weight loss and Tafel polarization test results approved the high efficiency of guar gum corrosion inhibition for carbon steel. In addition, Messali, et al. [22] employed guar gum as an effective non-toxic inhibitor for steel corrosion in a phosphoric acid medium. They reported that guar gum is a non-toxic and biocompatible polysaccharide with high corrosion inhibitory properties in phosphoric acid solutions.

The solution inside the concrete pores acts as an electrolyte. Furthermore, carbon dioxide and chloride ions can be introduced into the concrete matrix and result in the corrosion of the steel rebar [23]. There are two types of regions on the surface of the steel rebar namely anodic and cathodic spots. During the corrosion process, the iron atoms are transformed into electrons and ferrous ions. The electrons migrate from an anodic area to a cathodic one. This results in the formation of ferric hydroxide, which has a greater specific volume compared to steel. It results in the formation of cracks, lowering the adhesion between the rebar and the matrix, and detachment of the rebar from the concrete, which declines the service life of the structure.

The presence of chloride ions in the environment can significantly cause the deterioration of the passive layer [24]. In other words, chloride ions react with Fe atoms and OH^-^ ions and result in the formation of Fe_2_O_3_.nH_2_O, Fe(OH)_3_, and FeO(OH). Afterward, the free chloride ions continue the same reaction cycle.

Corrosion inhibitors are employed to overcome this problem by penetrating the concrete pores and cracks and via capillary effect to form a protecting corrosion resistance layer. The extract of R. damascena contains biocompatible phytoconstituents including ketones, phenols, aldehydes, and alcohols (comprises aromatic functional groups), which can provide supplementary sites for the formation of the protecting layer, preventing the aggressive agents, such as Cl^−^ and O_2_, from interacting with the steel rebar surface and hindering the corrosion progression [25]. Compounds including esculetin, 4-hydroxy chalcone, octanal, di-n-octyl phthalate, and 2-(phenyl methylene) comprise aromatic groups and heteroatoms (O, S, and N), which tend to adhere and cover the steel surface. The organic inhibitors improve the corrosion resistance of the reinforcement by obstructing the electron transfer at the rebar/cement interface and invasion of corrosive ions [26,27,28].

The main concern regarding the migrating inhibitors is their limited diffusion depth, which affects their application. Moreover, few studies have dealt with the use of these inhibitors to provide the engineers with a suitable inhibitor type and concrete design. The aim of this investigation was to study the inhibitory efficiency of eight different types of corrosion inhibitors for plain carbon steel rebars in an alkaline environment in the presence of chloride ions to compare the corrosion rate of the rebars in the presence and absence of Arabic and guar inhibitors. The conclusions of this study provide guidelines for engineers and researchers to facilitate the selection and application of the inhibitors.

## 2. Materials and Methods

### 2.1. Inhibitors

**Figure 1:**
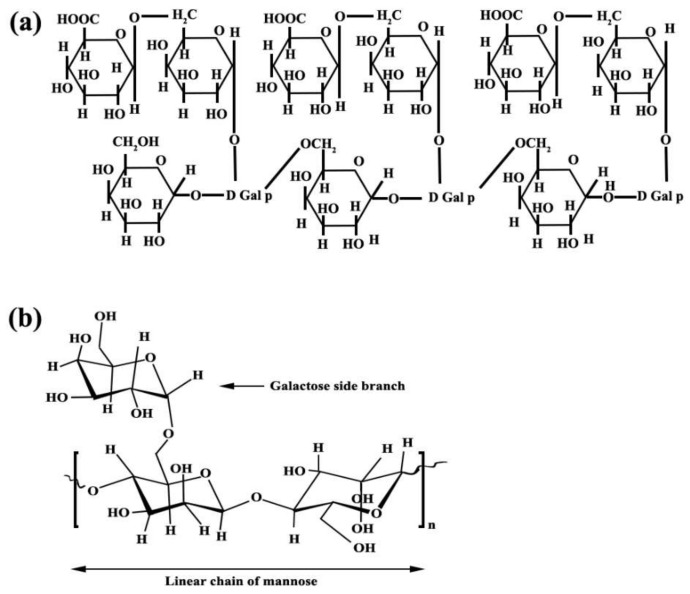
Chemical structure of (**a**) Arabic gum and (**b**) guar gum.

Arabic gum and guar gum with the structures of C_15_H_20_NNaO_4_ and C_10_H_14_N_5_Na_2_O_12_P_3_, respectively, with laboratory purity, were used as inhibitors and their chemical structures have been exhibited in Figure 1. Generally, two types of inhibitors, Arabic and guar gums, with 8 different compositions, have been used. The composition of each type of inhibitor and its properties are given in Table 1.

### 2.2. Concrete Preparation

In order to prepare concrete, a water/cement ratio of 0.5 with a combination of fine and coarse particles with a maximum dimension of 20 mm was used. Concrete samples were prepared based on an identical mixing scheme with a certain amount of aggregate, fine aggregates and the same amount of cement, the amount of water used according to Table 2. Corrosion inhibitors were first added to the water used to make concrete. To prepare concrete samples containing rebar, after setting, they were suspended in a solution containing 5 wt% of chloride ions and then used for corrosion tests. To increase the precision of the results, five samples from each inhibitor composition and the control sample were selected. The samples dimensions were 100 × 100 × 100 mm^3^ with a 10 mm steel bar at the center of the samples. Steel bars were ground with sandpaper and cleaned with acetone to remove the rust and contaminations.

### 2.3. Corrosion Behavior

**Figure 2:**
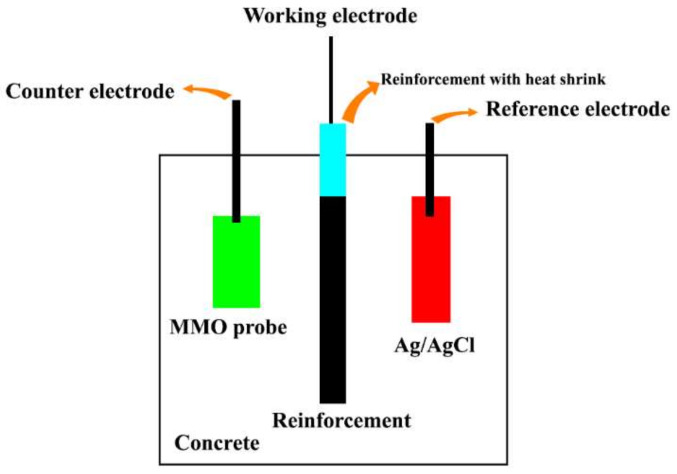
Reinforced concrete setups for electrochemical measurements.

The corrosion behavior of the reinforcing rebars in the concrete was determined via potentiodynamic polarization (PDP) and electrochemical impedance spectroscopy (EIS) analyses using an Autolab potentiostat. The PDP tests were carried out by applying a low perturbation potential (±20 mV) to the steel rebar at a scan rate of 1 mV/s to determine the variation in corrosion current and evaluate the polarization resistance ($R_{p}$). Then, the corrosion current density ($I_{corr}$) was calculated based on the $R_{p}$ value. Each sample was tested 1, 7, 28, 48, and 72 days after the immersion to determine the changes in the $R_{p}$ and $I_{corr}$ values. The obtained values were employed to analyze inhibitor efficiency compared to the control samples. On the other side, the EIS tests were carried out by applying an AC voltage of 5 mV over the frequency range of 100,000–0.01 Hz. Each sample was tested after 1, 7, 28, 48, and 72 days to determine the electrochemical values over time. Furthermore, obtained values were employed to analyze inhibitor efficiency compared to the control samples. Figure 2 shows the reinforced concrete setups for electrochemical measurements. The efficiency of each inhibitor’s composition was calculated by Equation (1) [19]:

(1) $$Inhibitor\;efficiency\;\left(\%\right)=\frac{{\left(I_{corr}\right)}_{intial}-{\left(I_{corr}\right)}_{final}}{{\left(I_{corr}\right)}_{intial}}\times 100$$

### 2.4. Water Absorption Test

The water absorption of the concrete was measured according to the BS 1062-3 protocol.

### 2.5. Electrical Resistivity of Concrete

The technique of the Wenner four-probe was employed to determine the surface resistivity of the concrete samples according to the AASTHO T358-15 standard [29]. After curing the samples dipped in water for 28 days, the surfaces of the samples were dried and they were placed in a container with 25 mm of water. Afterward, five tests were carried out for each sample at 90°. The resistivity meter probes were set along the longitudinal axis of the sample and attached to the concrete surface. Then, the average value of the tests was calculated. The tests were replicated every 14 days in the same conditions for each sample.

### 2.6. Characterizations

PerkinElmer Fourier transform infrared spectroscopy (FTIR, JASCO model FT/IR-6300, Yokohama, Japan) was performed in a range of 4000–400 cm^−1^. Furthermore, scanning electron microscopy (SEM, FEI model Quanta200, Hillsboro, OR, USA) and EDS analysis were used. Microscopic images and EDS analysis are taken from the interface of concrete and rebar (rebar side).

## 3. Results and Discussion

### 3.1. FTIR

**Figure 3:**
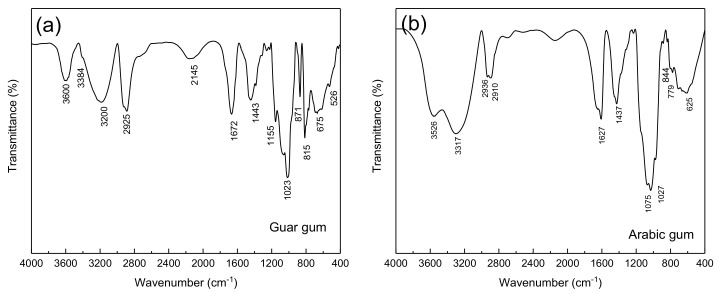
FTIR spectrum of the (**a**) gaur gum and (**b**) Arabic gum.

The guar gum FTIR spectrum is illustrated in Figure 3a. The bands at 1023, 2925, and 3384 cm^−1^ were related to the stretching vibration of C–O, C–H, and O–H groups, respectively. While the absorption bands at 1155 and 1443 cm^−1^ were related to the C–O–C group and bending of the C–H bonds, respectively.

In the Arabic gum FTIR spectrum shown in Figure 3b, the absorption bands in the ranges of 2800–3000 and 3000–3600 cm^−1^ were attributed to C-H and O-H stretching bonds, respectively. Furthermore, the peak at 3526.35 cm^−1^ was allocated to the presence of O-H bonds. Where the major adsorption peaks at 2910.87 and 2909.39 cm^−1^ corresponded to the C-H bond vibrations. COOH (carboxylic) groups were also detected at 625.4, 1436.91, 1437.2, and 1627.4 cm^−1^. On the other side, carboxylic acid functional groups were characterized by in-plane O-H bending peak at 1430 cm^−1^ [30]. Additionally, the peak at 1437.2 cm^−1^ can be caused by the presence of the symmetric stretches of COOH groups. The absorption bands in the range of 800–1200 cm^−1^ comprise the stretching of C-C, C-O, and C-O-C bonds in addition to the C-O-H bonds and C-H bending motions [31]. Furthermore, the peak at 779.2 cm^−1^ was related to both 1–6 linkage of mannose and 1–4 linkage of galactose [30]. On the other hand, the bands at 844 and 1027.62 cm^−1^ in the Arabic gum spectrum can correspond to arabinogalactan. It should be noted that the peaks in the range of 400–700 cm^−1^ were assigned to the pyranose ring skeletal modes of vibrations.

### 3.2. Compressive Strength

**Figure 4:**
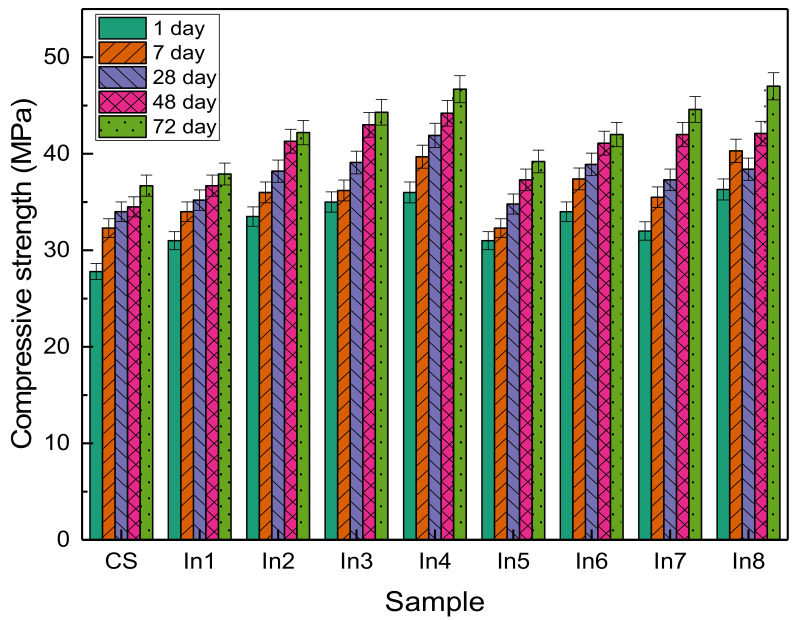
Compressive strength of concrete samples prepared with different inhibitors.

The compressive strength of concrete samples prepared with inhibitor compounds is presented in Figure 4. It can be seen that in all samples, the compressive strength has increased with age. Additionally, the use of inhibitors, in general, has increased the compressive strength compared to the sample without inhibitors. This has been observed in the study of other researchers [32,33,34,35,36]. Inhibitors can be used as an accelerator, which is characterized by higher hydration at an early age [37,38,39].

### 3.3. EIS Measurements

**Figure 5:**
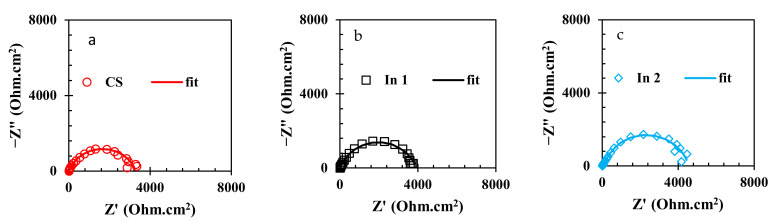

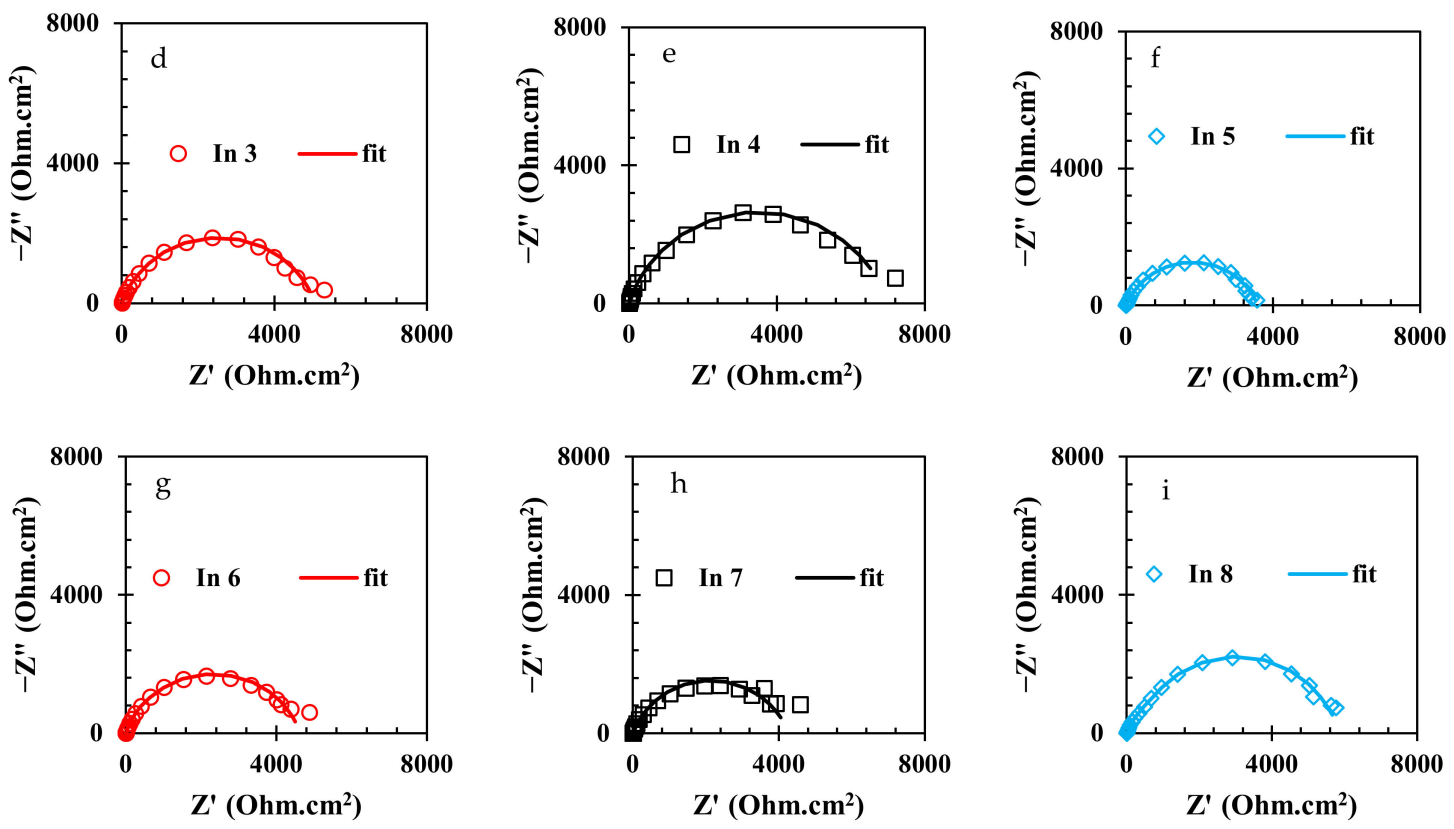
Nyquist curves of the rebars immersed in the electrolyte with/without the presence of inhibitors: (**a**) CS, (**b**) In1, (**c**) In2, (**d**) In3, (**e**) In4, (**f**) In5, (**g**) In6, (**h**) In7, and (**i**) In8.

**Figure 6:**
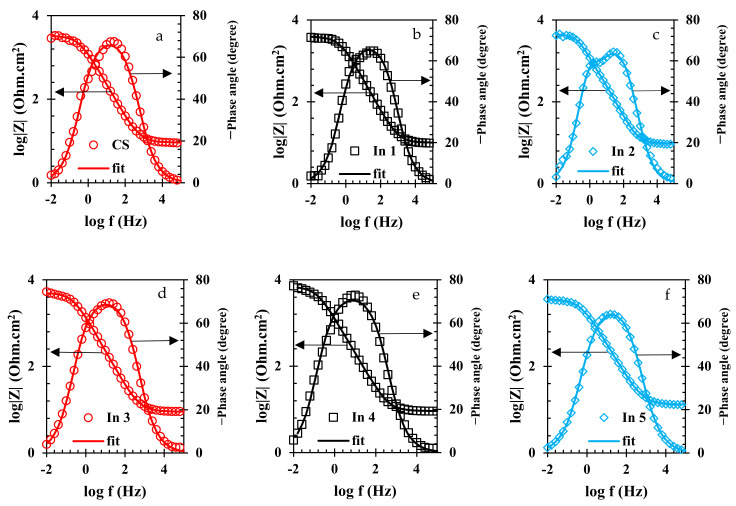

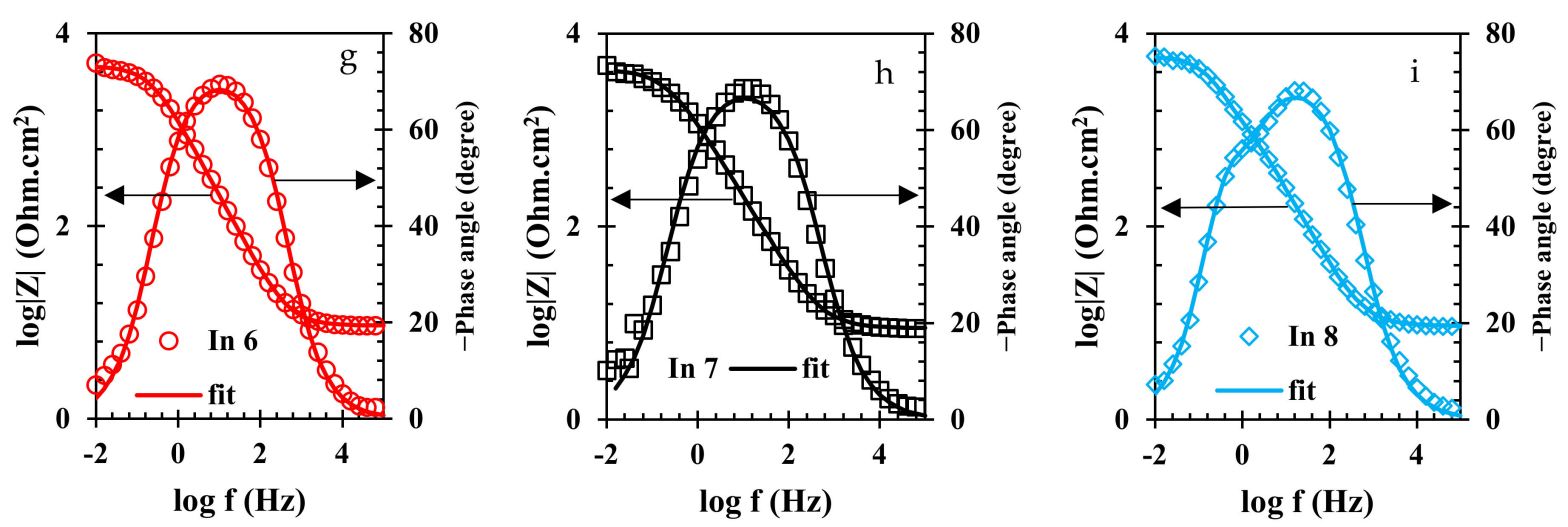
Bode-modulus and Bode-phase plots of the rebars immersed in the electrolyte with/without the presence of inhibitors: (**a**) CS, (**b**) In1, (**c**) In2, (**d**) In3, (**e**) In4, (**f**) In5, (**g**) In6, (**h**) In7, and (**i**) In8 samples.

**Figure 7:**
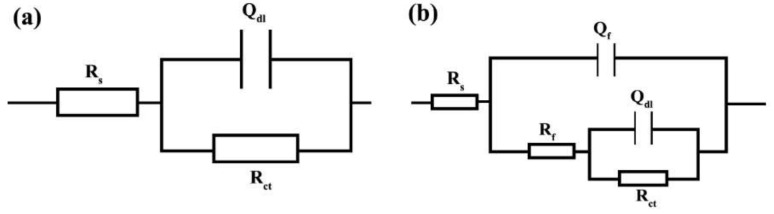
Modelling the EIS data by electrical equivalent circuits for: (**a**) single-time-constant and (**b**) two-time-constant.

To evaluate the electrochemical properties of the samples, the EIS analysis was used and the Nyquist and Bode curves of the under-studied samples are shown in Figure 5 and Figure 6, respectively. It is well known that the Nyquist diameter value, as well as the impedance at the lowest frequency (|Z| _0.01 Hz_), can be considered the indicator for total resistance against corrosion reactions [40]. Based on these indicators, it is clear that the In7 sample, as well as the In8 and In2 samples, have the highest values of Nyquist diameter and impedance at the lowest frequency among the under-studied samples, indicating the highest corrosion resistance in these samples. In addition, according to the indicators, the lowest corrosion resistance values belong to the In1 and In5 samples. Modeling of the EIS data on electrochemical equivalent circuits (EECs) is a popular technique for quantitative analysis of the EIS results [41]. As is illustrated in Figure 6, only one peak can be seen in the Bode-phase angle curves for all samples except for In2 and In8 which their Bode-phase angle curves consist of two overlapped peaks. Therefore, the two-time-constant EEC shown in Figure 7b was used to model the EIS data of In2 and In8 and one-time-constant EEC was employed for other samples (Figure 7a).

In these EECs, R_s_ is the solution resistance, R_f_ is the resistance of the film formed by adsorption of the inhibitors on the electrode surface, and Q_f_ represents the constant phase element (CPE) corresponding to the adsorbed film. Additionally, R_ct_ is the charge transfer resistance and Q_dl_ represents the constant phase element corresponding to the electrical double layer. In these circuits, CPE is used instead of the ideal capacitor to better fit the experimental data on the used EECs. The difference between a CPE and an ideal capacitor is in their impedance formula. The impedance formula of an ideal capacitor and a CPE is, respectively: Z_C_ = 1/jωC and Z_CPE_ = 1/(Y_0_jω)^n^, where C is capacitor capacity, ω is the angular frequency, Y_0_ is CPE admittance, and j represents √(−1). Therefore, if n = 1, CPE converts to an ideal capacitor, and if n = 0, it represents an ideal resistance.

Equivalence of the modeled samples with the EECs was performed by ZsimpWin software and the fitted curves are shown in Figure 5 and Figure 6. As can be seen in these figures, the modeled data have been properly fitted on the Nyquist and Bode curves obtained from experimental data. The parameter values obtained from the modeling are reported in Table 3. In Table 3, the value of R_t_ is calculated from the sum of the charge transfer resistance and the adsorbed film resistance. Furthermore, the inhibition efficiency (% IE) values are obtained from Equation (2).

%IE = 100 × (R_t_^in^ − R_t_^0^)/R_t_^in^ (2)

where R_t_^in^ and R_t_^0^ are the total resistance in the presence and the absence of the inhibitors, respectively. Additionally, the capacitance of the adsorbed film (C_f_) and the double layer (C_dl_) were calculated using Equations (3) and (4), respectively.

C_f_ = (Q_f_ × R_f_^1−n^)^1/n^ (3)

C_dl_ = (Q_dl_^1/n^ × ((R_s_ × R_ct_)/(R_s_ + R_ct_))^(1−n)/n^) (4)

According to Table 3 and as previously observed in the results for the Nyquist and Bode curves, the highest values of the inhibition efficiency and total resistance were possessed in In4, followed by In8 and In3 samples. On the other hand, by replacing the inhibitor molecules instead of water molecules and increasing the thickness of the adsorbed film on the immersed electrode, the values of C_C_ and C_dl_ increase [42]. According to Table 3, the lowest C_dl_ values belong to In4, In8, and In3 samples, indicating that the thickness of the formed layer on the immersed electrode in the presence of these inhibitors is thicker than those formed for other samples. In other words, in the presence of 250 A + 750 G, the thickness of the formed film on the immersed CS electrode was more than other inhibitor compounds and so the In4 sample is optimal in terms of corrosion inhibition performance among the under-studied samples. After this sample, the systems containing 1000 A + 1000 G (In8) and 250 A + 500 G (In3) inhibitor compounds showed the higher inhibition performance among the under-studied samples. 

### 3.4. Polarization Analysis

**Figure 8:**
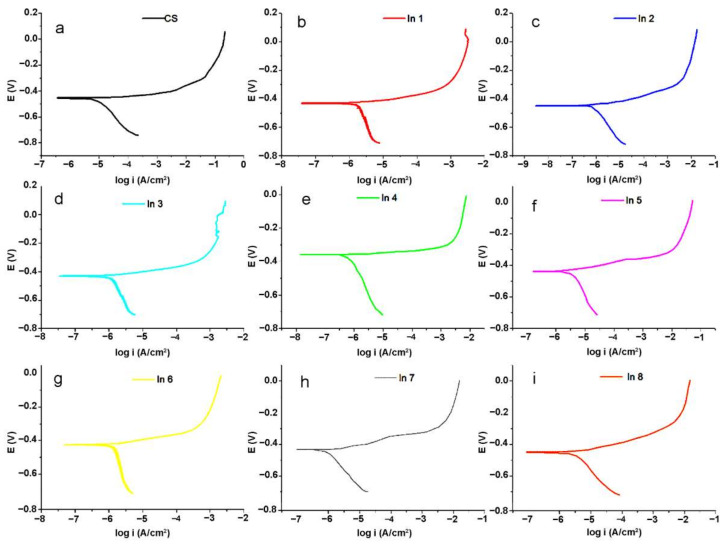
Potentiodynamic polarization plots of the electrode immersed in the electrolyte without and with the inhibitor compounds: (**a**) CS, (**b**) In1, (**c**) In2, (**d**) In3, (**e**) In4, (**f**) In5, (**g**) In6, (**h**) In7, and (**i**) In8 samples.

Potentiodynamic polarization analysis can provide more information about the inhibition mechanisms of the used inhibitor compounds. The obtained polarization curves for the under-studied samples are shown in Figure 8.

Generally, the Tafel extrapolation method is used to extract the electrochemical parameter values from the polarization curves [43]. The obtained electrochemical parameters from the Tafel extrapolation technique are tabulated in Table 4.

In Table 4, βc is the cathodic branche slop. E_corr_ is corrosion potential and i_cor_ is corrosion current density. The corrosion inhibition (%IE) values are obtained using Equation (5).

%IE = 100 × (i_corr_^0^ − i_corr_^in^)/i_corr_^0^ (5)

where i_corr_^in^ and i_corr_^0^ are the total resistance in the presence and the absence of the inhibitors, respectively. In this table, the values of anodic branch slop were not reported due to the active–passive behavior of the branch, as can be seen in Figure 8. From Table 4, it is clear that the addition of the inhibitor compounds into the solution led to a decrease in the corrosion current density, which decreased the corrosion rate of the immersed CS in the corrosive media. Among the inhibitor-containing samples, the highest and the lowest %IE are, respectively, obtained for In4 (250 A + 750 G) and In1 (100 A + 100 G). After In4, In8 (1000 A + 1000 G) and In3 (250 A + 500 G) possessed higher %IE values among the under-studied samples, that are in good agreement with the EIS results. In fact, it seems that by increasing the concentration of the G component in the inhibitor compounds up to 750 ppm, the inhibition performance of the compound improved. In the presence of more concentration of the G component (1000 ppm), the inhibition efficiency reduced slightly. On the other hand, lower concentration of the G component in the In1 (100 A + 100 G), In5 (500 A + 250 G), and In2 (250 A + 250 G) samples led to a decrease in the inhibition performance of the systems. It is noteworthy that the concentration of the A component did not have a significant effect on the inhibition performance of the compounds; thus, In5 with 500 ppm of the A component had a lower inhibition efficiency than In2 with 250 ppm of the A component. Furthermore, the variations in E_corr_ values did not have a specific trend for different samples, indicating that the inhibition compounds displayed a mixed-type inhibition mechanism. According to previous studies [21,44,45], the chemical compounds in guar gum and Arabic gum can be adsorbed on the metal surface through the donor–acceptor interaction between free electron pairs of heteroatoms as well as π-electron system from the aromatic rings and vacant d-orbital of the metal. So, it is expected that the inhibition performance of the inhibitors increases by increasing the concentration of the compounds in the corrosive electrolyte. However, an increase in viscosity occurs when the concentration of inhibitors exceeds a certain value. With increasing concentration, molecular chains, especially in polysaccharides, become closer to each other and as a result they become entangled [46]. This entanglement increases the viscosity, leading to an accumulation and lack of homogeneity. This uneven distribution of the inhibitors in the corrosive media reduces the effectiveness of inhibitors. On the other hand, these inhibitors have been added to a mixture of sand and cement, and they may be adsorbed in certain concentrations on these materials. Therefore, the non-linear behavior of the inhibition performance of the inhibitors with their concentration can be related to the complex phenomena occurred in the under-studied system. This nonlinear behavior for inhibitors has been previously observed in other studies of guar gum and other inhibitors [5,47].

### 3.5. Water Absorption

**Figure 9:**
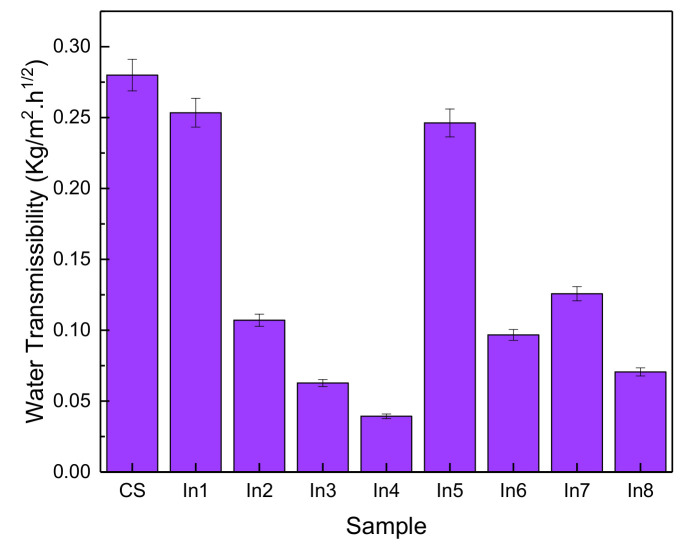
Water transmissibility of concrete samples prepared with different inhibitors.

Figure 9 shows the rate of water transmissibility as an indicator of permeability for the control sample and the samples with different inhibitory concentrations. It is observed that the use of inhibitors in the ln3, ln4, and ln8 samples has reduced the water transfer rate by about 86, 77, and 75%, respectively. According to BS EN 1062-1: 2004, they are classified as “W3”, i.e., coatings with low permeability. In general, water transmissibility has been reduced with the use of inhibitory compounds. In1 and In5 samples showed a decrease in water transfer compared to the reference sample of 9% and 12%, respectively, and other samples showed a decrease in water transmissibility of about 55 to 65%. Therefore, it can be said that the use of inhibitors has a significant effect on corrosion resistance [48,49].

### 3.6. Electrical Resistivity

**Figure 10:**
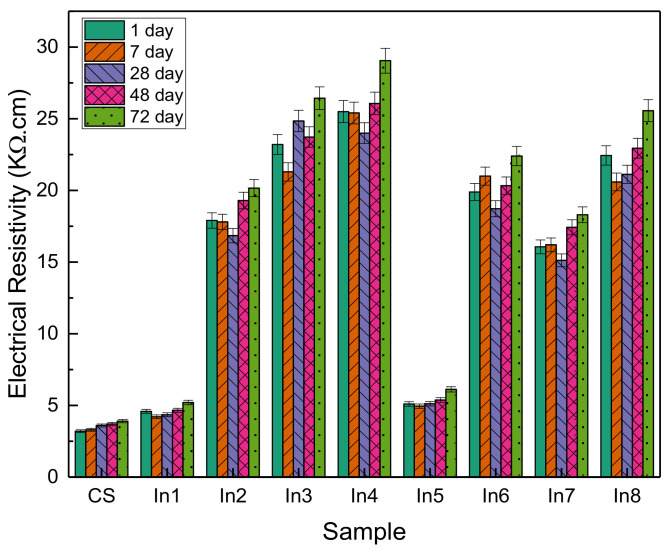
Electrical resistance of concrete samples prepared with different inhibitors.

Figure 10 shows the changes in electrical resistance of the samples at 1, 7, 28, 48, and 72 days. As can be seen, with increasing time, the trend of changes in electrical resistance has been increasing, although the increase has not been large. In the control sample, the electrical resistance was in the range of 3.2 to 3.9 ohms and did not change significantly with increasing time. The addition of barrier compounds to concrete in the In1 sample did not increase much compared to the control sample and was in the range of 4.2 to 2.5 ohms. With the increasing concentration of the inhibitors, a significant increase in electrical resistance is observed, which in the In3 and In4 samples has changed in the range of 24 to 29 and 21 to 26 ohms, respectively. In the In5 sample, as in the In1 sample, the change was very small. In general, it indicates an increase in the electrical resistance of concrete using appropriate concentrations of inhibitors. Increasing the electrical resistance of concrete represents greater resistance of concrete to corrosion and greater protection against water [50].

### 3.7. Microstructure

**Figure 11:**
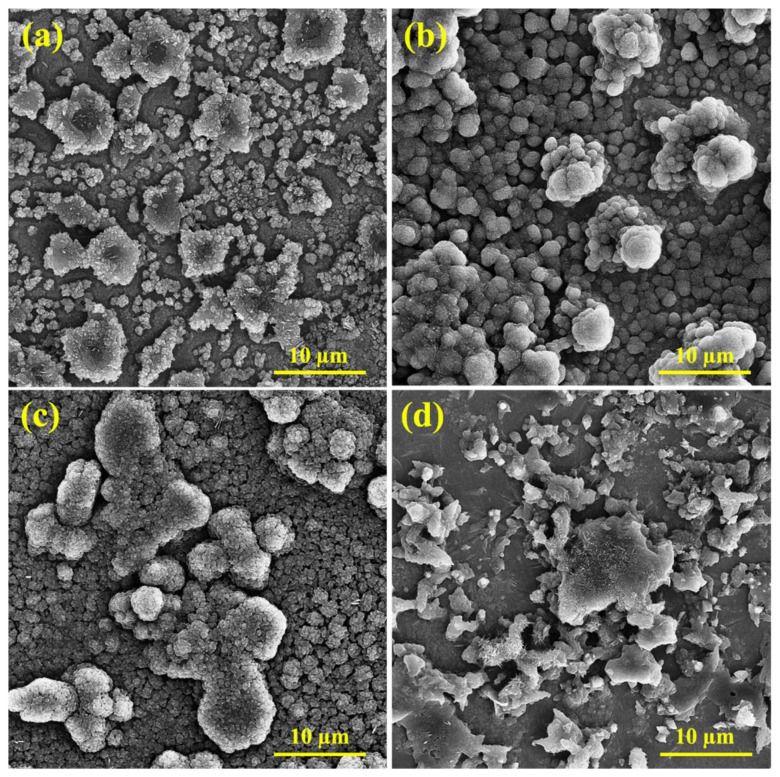
SEM micrographs of the samples: (**a**) CS, (**b**) In1, (**c**) In4, and (**d**) In8.

Figure 11 illustrates the effect of the presence of inhibitors on the microscopic structure of the CS, In1, In4, and In8 samples after corrosion tests. As shown in Figure 11a, severe corrosion is evident due to the lack of inhibitors in the acidic environment. Moreover, the corrosive products, which were adhered to the surface, apparently damaged the surface and caused inhomogeneity. In steel anodes, the attacks of corrosion are typically related to high carbon regions. Attacks preferably initiate from the grain boundaries and then extend from the dendritic zones into the grains. Hence, corrosion begins in the inter-granular areas. This is attributed to the accumulation of alloying elements in the inter-granular areas. The alloying elements have a high reactivity; therefore, their improper distribution in the alloy matrix has caused non-uniform corrosion of the matrix. The SEM image of the CS sample in Figure 11a presents parallel grooves in the corroded areas. By comparing the SEM micrographs, it is evident that in the absence of inhibitors the sample has an acicular microstructure that overcomes the cauliflower microstructure, while in the level absorbed by the inhibitor the cauliflower morphology increases, and the acicular structure is very limited. The microscopic observations of the samples demonstrated that the phase distribution, grain size, and morphology of the steel rebars affected their corrosion behavior. Figure 11b,d show the inhibitor distributions on the microscopic structures of the In1 and In8 samples after corrosion test. The inhibitor-adsorbed sample revealed a smoother surface with an entirely different morphology due to the formation of a protective film by the adsorbent inhibitor. The inhibitor reduced the corrosion rate at the anode surface and caused more uniform corrosion. However, the agglomeration of inhibitors can be seen in the image, which is a mixture of inhibitors due to increased viscosity. This distribution of inhibitors, although covering the surface of the sample, causes non-uniform corrosion of the surface. However, the sample surface containing the inhibitor In4 shows more homogeneous distributions with smaller particle sizes, which could cover a large area of the surface with higher density. SEM micrographs in Figure 11c confirmed the proper coverage of the surficial passive film, which acted as a barrier to the aggressive ions. This has reduced the corrosion rate of the sample compared to other samples and confirmed the high inhibition efficiency of guar and Arabic gum combination in acidic environments.

**Figure 12:**
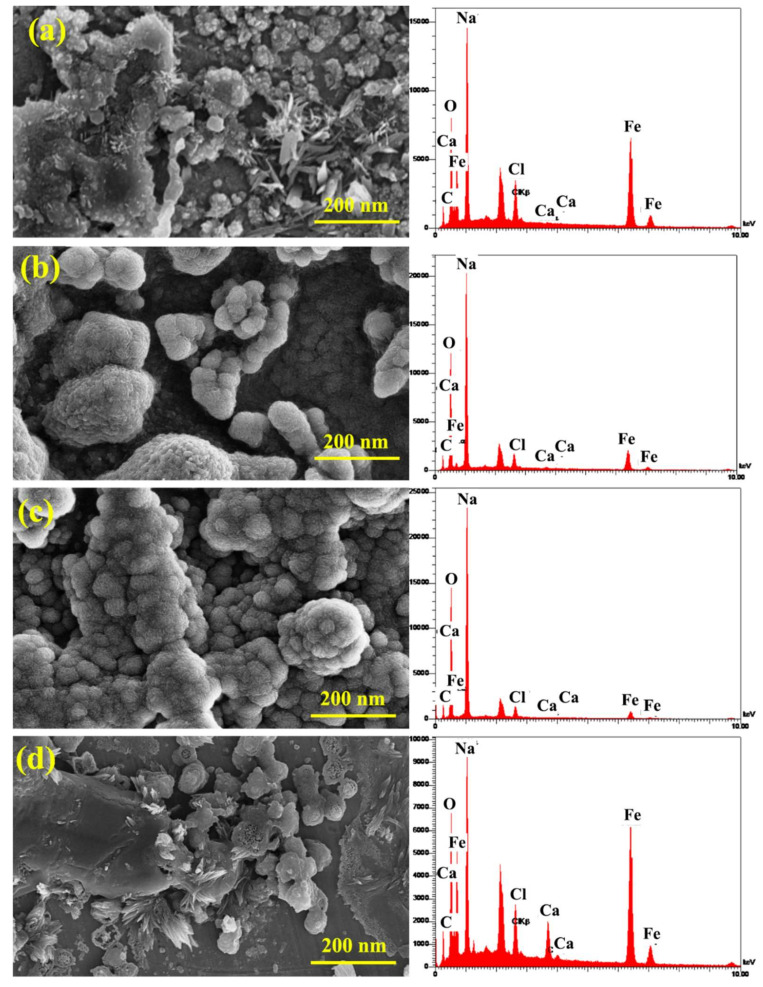
SEM micrographs and EDS analysis of the samples: (**a**) CS, (**b**) In1, (**c**) In4, and (**d**) In8.

Figure 12 displays the grain size of the sample in the absence/presence of the inhibitors, which shows that the inhibitors enhance the grain size, indicating the suitable inhibitor adsorption on the sample surface. Table 5 and Figure 12 present the elemental analysis of the samples and show that the contents of C, Cl, Ca, and Fe in the solutions containing the inhibitor decrease compared to the CS sample. This resulted in the reduction in corrosion in the presence of the inhibitor that shows the increased percentages of O and Na. The lack of the inhibitors caused the sample surface to have higher non-uniformity. Accordingly, the concentration gradient reduced and caused an improved alloying element distribution; therefore, the electrochemical properties became uniform throughout the anode surface. In other samples, incompatible inhibitor distribution caused the non-uniform corrosion. By simultaneous addition of both increasing inhibitors, the surface adsorption improved properly, and additionally, the grain size and distances between the grains increased, and suitable distribution of the inhibitor combination was obtained. Hence, adequate inhibitor adsorption on the reinforcement reduced the non-uniform and spontaneous corrosion. The elemental point analyses presented in Figure 12b,c show the occurrence of the phase containing of In1 and In4 samples, which indicates uniform corrosion of the anode by the inhibitor combination. The compounds of the inhibitor were adsorbed on the reinforcement surface and reduced the detrimental effects of chloride ions on the steel and increased the service life of the rebar in the environment. According to the elemental point analyses, it can be concluded that the formed halos represent the corroded areas in the simulated concrete environment. Moreover, bold white spheres are evident on the halos, which have formed due to the simultaneous presence of guar and Arabic gums.

**Figure 13:**
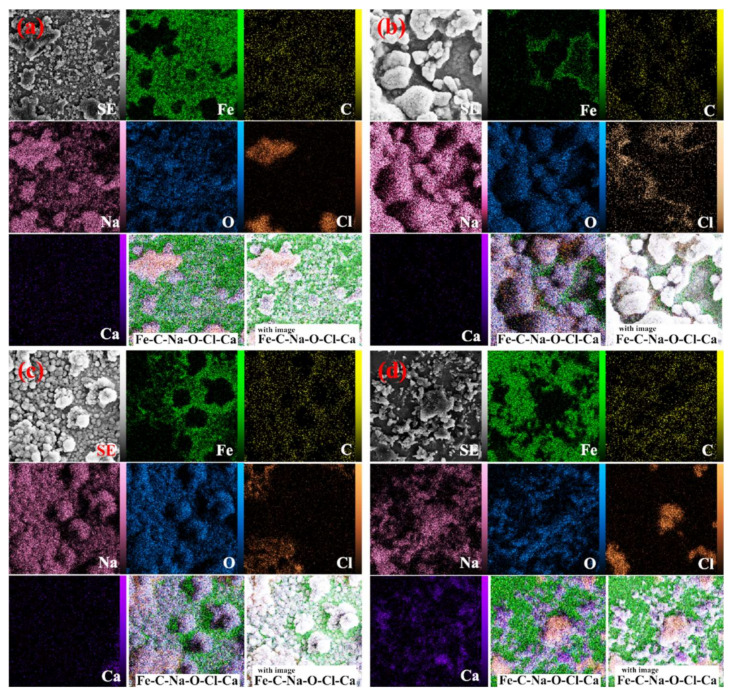
Elemental map analysis of the sample: (**a**) CS, (**b**) In1, (**c**) In4, and (**d**) In8.

Figure 13 demonstrates the elemental mapping analyses of the control sample and the ones containing the inhibitors. The figures confirmed how the inhibitors were distributed on the sample surface. Moreover, it can be seen that the inhibitor distribution on the In7 sample surface is more uniform than In4 sample. To verify the arguments above, elemental point analysis was carried out on the formed white halos as well as in distance of the halos (Figure 13). By comparing the elemental point analyses, it was seen that when enhancing the inhibitor concentration, the adsorption on the reinforcement increased to a specified level and then decreased by further enhancement of the inhibitor content. It could be concluded that non-uniform corrosion was observed in the CS sample, which is due to the absence of inhibitors and inhibitory mechanisms. The surficial adsorption of the inhibitors was observed for the samples in the solutions containing inhibitors. Furthermore, as expected, by corresponding the elemental point analyses to the SEM micrographs, formation of a uniform layer with the lowest detachment was seen in these samples. In other words, the concentration gradient was decreased and the distribution of the inhibitors was improved. Consequently, the electrochemical properties were uniform throughout the anode. In the CS sample, the lack of inhibitors on the steel matrix caused non-uniform corrosion. Nevertheless, the grain size of the sample in the solution containing both inhibitors increased and the distribution of inhibitors was more uniform on the sample surface. The variation in the adsorption level implied the repletion of inhibitors at the grain boundaries and inter-granular regions. Furthermore, the accumulation altered the chemical composition uniformity in different regions of the anode. The electrochemical responses of the regions changed, increasing the occurrence of non-uniform and spontaneous corrosions. 

### 3.8. FTIR

**Figure 14:**
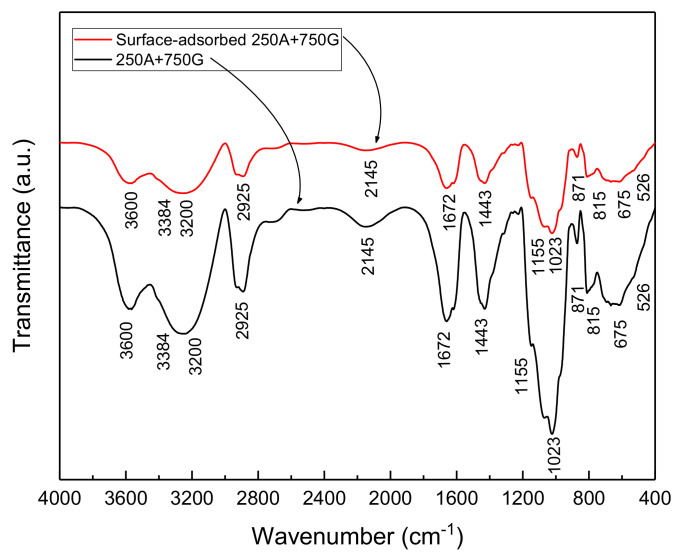
FTIR spectrum of the combination of the In7 sample and In7 inhibitor adsorbed on the metal surface.

Figure 14 shows the FTIR spectra of In7 and the surface of the reinforcement, in contact with the In7 sample. Based on the spectrum of combination of In7, all peaks related to the guar gum and Arabic gum are evident. This indicates that a mixture of the two inhibitors has formed a protective layer on the rebar surface. The absence of new peaks indicates that the inhibitor neither reacts with the surface nor forms a new composition. It can be seen that most of the guar gum peaks are more intense and cover the Arabic gum peaks, which is normal due to its higher amount. In the spectrum related to the adsorbed compound on the surface of the metal, it can be observed that the intensity of all peaks has decreased. Some authors have reported that the reduction in peak intensity observed near the 3300 cm^−1^ wavenumber may be due to the binding of some hydroxyl groups to the metal surface through the formation of hydrogen bonds [20,51]. The decrease in peak intensities in the range of 1430 and 1630 to 1690 cm^−1^ can also be related to the hydrogen bond formation between the carboxylic groups in the compounds and the surface of the oxidized metal, as other researchers have reported [20,22,51,52]. Other researchers have attributed the reduction in peak intensities in the range of 800 to 1150 cm^−1^ to the possible interaction between the intra-ring oxygen atom of the galactose unit Arabic gum and the C6-O of the monosaccharide unit with the surface [22,51,53].

## 4. Conclusions

In the current research, eco-friendly inhibitors were selected to investigate their effect on the chloride-induced corrosion of reinforcement in concrete. Therefore, nine types of Arabic and guar gums combinations with 100, 250, 500, 750, and 1000 ppm were added to the concrete for 1, 7, 28, 48, and 72 days. To investigate the concrete behavior in the presence of inhibitors, corrosion behavior, water transmissibility, electrical resistivity, and compressive strength of concrete were investigated.

1. It is shown that Arabic and guar gums are effective inhibitors for reinforced concrete structures. Furthermore, FTIR and SEM analyses show that by enhancing the inhibitor concentrations, the grain size of the inhibitor layers enhances and showed that the suitable inhibitory adsorption on the reinforcement surface was performed. Furthermore, results showed that a suitable inhibitor distribution on the reinforcement was achieved, which caused desirable cathode current.
2. The compressive strength has increased with age and also the use of an inhibitor in general has increased the compressive strength compared to the sample without an inhibitor.
3. According to the diagram, the highest inhibitory efficiency was related to the In4 sample, followed by the In3 and In8 samples.
4. Water transmissibility has been reduced with the use of inhibitory compounds. In1 and In5 samples showed a decrease in water transfer in them compared to the reference sample of 9% and 12%, respectively, and other samples showed a decrease in water transmissibility of about 55 to 65%. Therefore, it can be said that the use of inhibitors has a significant effect on corrosion resistance.
5. Generally, it indicates an increase in the electrical resistance of concrete using appropriate concentrations of inhibitors. Increasing the electrical resistance of concrete represents greater resistance of concrete to corrosion and greater protection against water.
6. Two samples, In1 and In5, had a higher corrosion rate than the control sample. This can be due to the lack of proper mixing between the gums and the lack of proper surface coverage, which has caused severe corrosion in the samples by uneven distribution on the surface. Corrosion rates for other inhibitor compounds have decreased over time. Sample In4 had the best performance, which had a deterrent effect of reducing the corrosion rate by nearly 50%. In general, optimum results were obtained in samples with higher concentrations of Arabic gum or equal concentrations of Arabic gum and guar gum, and lower efficiency was seen in samples with higher concentrations of guar gum.

## Figures and Tables

**Table 1:** The dosage and grain size of inhibitor and pH.

Inhibitor	Arabic Gum (ppm)	Guar Gum (ppm)	pH	Grain Size (nm)
CS	-	-		-
In1	100	100	6.4	17 ± 1
In2	250	500	6.3	20 ± 2
In3	500	250	5.8	19 ± 2
In4	250	250	6.3	18 ± 1
In5	500	500	6.4	29 ± 2
In6	750	250	5.3	32 ± 2
In7	250	750	6.4	34 ± 3
In8	1000	1000	6.5	46 ± 3

**Table 2:** The composition of the concrete.

Cement	Stone Powder	Grit	Sand	Water
333 (g)	167 (g)	625 (g)	708 (g)	120 (g)

**Table 3:** Electrochemical parameters from modeling calculations.

Sample	R_s_ (Ω·cm^2^)	CPE_c_, Y_0_ (S·sec/cm^2^)	n_1_	C_c_ (μF/cm^2^)	R_c_ (Ω·cm^2^)	CPE_dl_, Y_0_ (S·sec^n^/cm^2^)	n_2_	C_dl_ (μF/cm^2^)	R_ct_ (Ω·cm^2^)	Rt (Ω·cm^2^)	%IE
CS	9.13 ± 1.11	-	-	-	-	1.65 × 10^−4^ ± 2.76 × 10^−5^	0.79 ± 0.02	29.31 ± 1.39	3288 ± 134	3288 ± 134	-
In1	10.76 ± 1.30	-	-	-	-	1.74 × 10^−4^ ± 1.11 × 10^−5^	0.78 ± 0.04	29.58 ± 1.26	3568 ± 187	3568 ± 187	7.85 ± 1.58
In2	9.33 ± 1.89	1.45 × 10^−4^ ± 1.54 × 10^−5^	0.79 ± 0.01	98.44 ± 1.33	1607 ± 122	1.73 × 10^−4^ ± 2.06 × 10^−5^	0.77 ± 0.03	25.28 ± 1.98	2959 ± 165	4566 ± 156	27.99 ± 1.88
In3	9.05 ± 1.34	-	-	-	-	9.15 × 10^−5^ ± 7.13 × 10^−6^	0.81 ± 0.04	17.31 ± 1.49	5053 ± 199	5053 ± 199	34.93 ± 2.98
In4	9.15 ± 1.29	-	-	-	-	7.16 × 10^−5^ ± 2.32 × 10^−6^	0.83 ± 0.05	15.96 ± 1.54	69.83 ± 211	6983 ± 211	52.91 ± 3.45
In5	9.67 ± 1.43	-	-	-	-	1.77 × 10^−4^ ± 9.70 × 10^−6^	0.78 ± 0.03	29.34 ± 1.37	3918 ± 209	3918 ± 209	16.08 ± 3.99
In6	9.27 ± 1.33	-	-	-	-	1.17 × 10^−4^ ± 1.98 × 10^−5^	0.81 ± 0.04	23.46 ± 1.89	4620 ± 176	4620 ± 176	28.83 ± 2.78
In7	8.83 ± 1.29	-	-	-	-	1.61 × 10^−4^ ± 7.75 × 10^−6^	0.79 ± 0.04	28.17 ± 1.44	4258 ± 219	4258 ± 219	22.78 ± 3.98
In8	9.39 ± 1.21	1.40 × 10^−5^ ± 2.26 × 10^−6^	0.81 ± 0.03	6.55 ± 1.34	2814 ± 238	1.02 × 10^−4^ ± 2.70 × 10^−5^	0.79 ± 0.05	16.12 ± 1.87	3030 ± 215	5844 ± 221	43.74 ± 5.34

**Table 4:** The electrochemical parameters obtained using the Tafel extrapolation method.

Sample	β_c_ (V·dec^−1^)	E_corr_ (V)	i_corr_ (μA/cm^2^)	%IE
CS	0.231 ± 0.03	−0.461 ± 0.08	2.51 ± 0.14	-
In 1	0.162 ± 0.03	−0.465 ± 0.07	2.50 ± 0.17	11.62 ± 1.30
In 2	0.204 ± 0.02	−0.452 ± 0.06	2.47 ± 0.24	29.61 ± 2.8
In 3	0.304 ± 0.04	−0.43 ± 0.08	2.43 ± 0.19	42.10 ± 4.87
In 4	0.337 ± 0.05	−0.361 ± 0.04	1.51 ± 0.21	53.70 ± 3.45
In 5	0.305 ± 0.03	−0.432 ± 0.05	2.49 ± 0.34	23.01 ± 2.55
In 6	0.33 ± 0.05	−0.425 ± 0.04	2.41 ± 0.30	32.79 ± 3.56
In 7	0.263 ± 0.03	−0.434 ± 0.05	2.48 ± 0.55	31.41 ± 5.09
In 8	0.177 ± 0.03	−0.46 ± 0.06	2.32 ± 0.54	47.65 ± 3.33

**Table 5:** EDS analysis of the samples.

Element	at%	wt%	at%	wt%	at%	wt%	at%	wt%
C	15.5	7.8	13.6	7.9	14.1	8.7	17.5	8.8
O	26.7	18.2	35.5	27.7	38.1	31.4	29.8	20.0
Na	41.7	40.8	44.4	49.7	44.3	52.4	33.2	32.0
Cl	5.4	8.2	2.8	4.9	2.2	4.1	5.1	7.7
Ca	0.6	1.0	0.5	1.0	0.2	0.4	3.3	5.5
Fe	10.1	24.0	3.2	8.8	1.1	3.0	11.1	26.0
Total	100.00	100.00	100.00	100.00	100.00	100.00	100.00	100.00

## Data Availability

Not applicable.
